# Pseudo-Four Component Synthesis of Mono- and Di-Benzylated*-*1,2,3-Triazoles Derived from Aniline

**DOI:** 10.3390/molecules19010055

**Published:** 2013-12-20

**Authors:** Daniel Mendoza-Espinosa, Guillermo E. Negron-Silva, Leticia Lomas-Romero, Atilano Gutierrez-Carrillo, Rosa Santillán

**Affiliations:** 1 Departamento de Ciencias Básicas, Universidad Autónoma Metropolitana-Azcapotzalco, Avenida San Pablo No. 180, C.P. 02200, México D.F., Mexico; E-Mail: dmesp@correo.azc.uam.mx; 2 Departamento de Química, Universidad Autónoma Metropolitana-Iztapalapa, Av. San Rafael Atlixco No. 186, C.P. 09340, México D.F., Mexico; E-Mails: llr@xanum.uam.mx (L.L.-R.); agrmn@xanum.uam.mx (A.G.-C.); 3 Departamento de Química, CINVESTAV-IPN, Apdo. Postal 14-740, 07000, México D.F., Mexico; E-Mail: rsantill@cinvestav.mx

**Keywords:** click chemistry, aniline, dibenzylated triazoles, catalysis

## Abstract

The pseudo-four component click synthesis of dibenzylated 1,2,3-triazoles derived from aniline is reported. The cycloaddition of sodium azide to *N*-(prop-2-ynyl)-benzenamine (**I**) in the presence of equimolar amounts of *p*-substituted benzyl derivatives, yields a mixture of mono- and dibenzylated 1,2,3-triazoles. When two equivalents of the benzyl derivative are added to the multicomponent reaction, the selective preparation of the dibenzylated compounds is achieved. The reactivity of the aniline N-H bond in monobenzylated 1,2,3-triazoles was tested by treatment with one equivalent of a *p*-substituted benzyl chloride at 40 °C, rendering the dibenzylated derivatives quantitatively.

## 1. Introduction

One-pot multicomponent reactions are increasingly important in areas such as organic chemistry and materials science because they offer several advantages over classical linear syntheses [1,2,3]. Multicomponent reactions are highly convergent, offering a notable increase in molecular complexity and topology in a single step [4,5,6,7,8,9]. Another important feature concerns the reduction of waste production as a result of the decrease of synthetic or isolation steps along with time savings [10,11,12,13,14].

Within the most conventional multicomponent processes, those based on the peculiar reactivity of isocyanides, such as the Doemling, Ugi and Passerini reactions are the most studied [15,16,17,18,19,20,21,22,23,24,25,26]. Since the copper(I) azide-alkyne catalyzed cycloadditon (CuAAC) was reported independently by the groups of Sharpless and Medal in 2002, a tremendous variety of 1,4-disusbtituted 1,2,3-triazoles have been prepared [27,28,29,30,31,32,33,34]. Due to the importance and interest of the 1,2,3-triazole motif in the drugs and pharmaceuticals design, there is continuous quest for the development of a simple and efficient methods for their preparation in one-pot multicomponent processes [35,36,37,38,39,40,41,42,43].

Most click processes for triazole synthesis are usually based on three component reactions [44,45,46,47,48]. Relatively few reports on four component click reactions have been reported so far [49,50,51,52]. As part of our ongoing program in triazole chemistry, we envisioned the synthesis of 1,2,3-triazoles as potential steel corrosion inhibitors and/or transition metal ligands. In the present report, we disclose a general approach for the one-pot synthesis of a series of mono-, and dibenzylated 1,2,3-triazoles based on aniline as the parent compound. The modular synthesis described herein allows for the preparation of *N*-benzylated 1,2,3-triazoles under mild conditions and the functionalization of the N-H bond in the aniline moiety simultaneously through a pseudo-four component click process.

## 2. Results and Discussion

### 2.1. Synthesis and Characterization

**Scheme 1:**
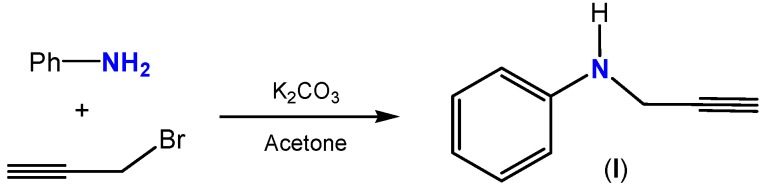
Synthesis of alkyne **I**.

The initial synthetic step involved the preparation of *N*-(prop-2-ynyl)benzenamine (**I**) by deprotonation of aniline with potassium carbonate in acetone at room temperature, followed by the addition of equimolar amounts of propargyl bromide. After work up and purification through column chromatography on silica gel, alkyne **I** was isolated in 79% yield according to Scheme 1 [53].

The click reaction between **I** and sodium azide was carried out in a 4:1 water/ethanol mixture using Cu(OAc)_2_ and sodium ascorbate as the reagents to render the Cu(I) active catalyst. After addition of benzyl chloride to the reaction mixture and stirring at room temperature for 18 h we observed the complete consumption of the alkyne. At our first intention, we had targeted the preparation of the *N*-benzylated 1,2,3-triazole **2a**, however, TLC analysis of the reaction mixture showed two main products. After separation by column chromatography using CH_2_Cl_2_ and CH_2_Cl_2_–MeOH (98:2) as gradient mixtures, the two products were isolated. The ^1^H-NMR spectra of the second fraction showed two single peaks corresponding to the methylene groups and a triplet signal assigned to the NH bond from the aniline moiety. All the patterns were consistent with the structure of the expected triazole **2a**. On the other hand, the spectrum of the first fraction displayed three different methylene moieties and the signal for the NH group no longer appeared. Careful analysis of the ^1^H and ^13^C spectra and the M+H [355.1923] ion of **1a** in HRMS (ESI-TOF) confirmed the dibenzylated triazole structure of this molecule. Thus, to our delight, the preparation of triazole **1a** resulted from a one-pot pseudo-four component click process.

The scope of the method was extended with the use of several *p*-halobenzylic derivatives yielding the mono- and di-benzylated 1,2,3-triazoles **1b**‒**e** and **2b**–**e**, respectively. Likewise the use of several benzylic derivatives allows for the preparation of 1,2,3-triazoles which feature several halogen substituents. As depicted in Table 1, the reaction proceeds smoothly under mild reaction conditions.

**Table 1:** Synthesis of mono- and dibenzylated triazoles. 
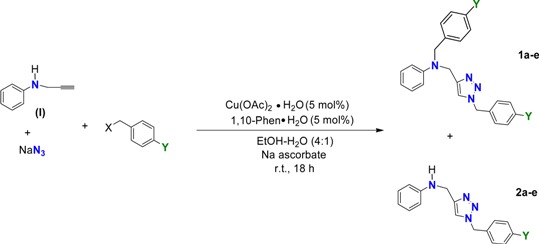 *Reagents and conditions*: Compound **I** (1.14 mmol), Cu(OAc)_2_·H_2_O (5 mol%), 1,10-phenanthroline (5 mol%), sodium ascorbate (1.14 mmol), sodium azide (1.40 mmol), *p*-substituted benzyl halogenide (1.40 mmol), stirring at room temperature for 18 h in EtOH/H_2_O (4:1).

Entry	X	Y	Product	Yield (%)	Product	Yield (%)
1	Cl	H	1a	42	2a	35
2	Cl	F	1b	40	2b	31
3	Cl	Cl	1c	39	2c	35
4	Br	Br	1d	42	2d	29
5	Br	I	1e	25	2e	42

Triazoles **1a**–**f** and **2a**–**f** were conveniently characterized by FT-IR, ^1^H and ^13^C-NMR spectroscopy, and also by High Resolution Mass Spectrometry. The formation of the 1,2,3-triazoles was apparent by the presence in the ^1^H-NMR of the characteristic singlet due to the triazolyl protons in the δ = 7.99–8.02 ppm for **1a**–**e** and δ = 7.98–8.01 ppm region for **2a**–**e**, respectively.

For most of the series, the click reaction provides the dibenzylated products in higher yields with the exception of the reaction with 4-iodobenzyl chloride in which the monobenzylated triazole is the major product. This may be related to a deactivating effect on the N-H bond due to the presence of the large iodo atom.

As the dibenzylated products were an interesting example of a pseudo-four component process, we focused next on optimization of their preparation. Considering that the dibenzylated products were favored in the original process, we envisioned that we could facilitate their selective preparation by increasing the equivalents of *p*-halogenated benzyl derivatives. Thus, the original process was optimized using 2.4 equivalents of the benzylated reactant and 24 h stirring at room temperature. As denoted in Table 2, the reaction proceeds nicely providing dibenzylated products **1a**–**e** in high yields.

**Table 2:** Synthesis of bis-1,2,3-triazoles **1a**–**e**. 
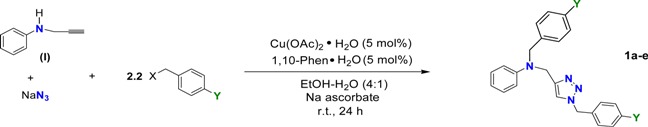 *Reagents and conditions*: Compound **II** (1.14 mmol), Cu(OAc)_2_·H_2_O (5 mol%), 1,10-phenanthroline (5 mol%), sodium ascorbate (1.14 mmol), sodium azide (1.40 mmol), *p*-substituted benzyl halogenide (2.80 mmol), stirring at room temperature for 24 h in EtOH/H_2_O (4:1).

Entry	X	Y	Product	Yield (%)
1	Cl	H	1a	71
2	Cl	F	1b	76
3	Cl	Cl	1c	80
4	Br	Br	1d	79
5	Br	I	1e	86

Once we had selectively prepared **1a**–**e**, we targeted the synthesis of dibenzylated compounds containing mixed benzyl moieties. With this aim, the reaction was carried out in the presence of one equivalent of *p*-iodobenzyl chloride and one equivalent of *p*-bromobenzyl chloride and stirring for 24 h showing by TLC the presence of four compounds in similar ratios. Analysis by of the ^1^H and ^13^C suggested the presence of two mono- and two benzylated compounds, however, due to the complexity of the mixture no separation was possible.

**Scheme 2:**
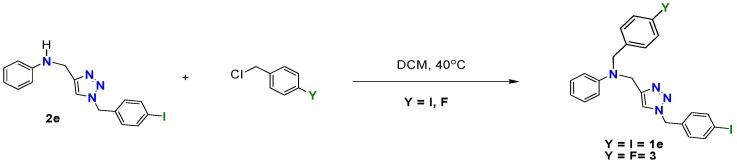
Synthesis of dibenzylated triazoles **1e** and **3**.

Knowing that the *N*-benzylated 1,2,3-triazoles contained a N-H bond, we decided to test its reactivity towards benzyl derivatives in order to obtain bifunctionalized species. Monitoring the addition of equimolar amounts of *p*-iodobenzyl chloride to triazole **2e** at room temperature in chloroform, it was observed that after 24 h, only 5% conversion to the desired benzylated aniline was reached. After several trials, we observed that carrying out the reaction at 40 °C and 12 h of stirring resulted in the full consumption of the starting material. After separation by column chromatography on silica gel with CH_3_Cl as eluent, compound **1e** was obtained as a waxy solid in 94% yield (Scheme 2). A second example of the N-H group functionalization of compound **2e**, was achieved by treatment with *p*-fluorobenzyl chloride under similar reaction conditions. Compounds **3** that displays different *p*-substituted-benzyl moieties, was obtained in 90% yield after work up and purification (Scheme 2).

All the reported 1,2,3-triazoles were obtained as clear solids and their robustness is denoted by their high stability in solution and under aerobic conditions.

## 3. Experimental

### 3.1. General Methods

Commercially available reagents and solvents were used as received. *N*-(prop-2-ynyl)benzenamine (**I**) was synthesized as reported in the literature [53]. Flash column chromatography was performed on Kieselgel silica gel 60 (230–400 mesh). Melting points were determined on a Fisher-Johns apparatus and are uncorrected. IR spectra were recorded on a Bruker Alpha FT-IR/ATR spectrometer. NMR spectra were obtained with a JEOL ECA-500 (500 MHz) spectrometer. Chemical shifts (δ) are given in ppm downfield from Me_4_Si as an internal reference; coupling constants are given in Hertz. High-resolution mass spectra (HRMS) were recorded on a JEOL JMS-SX 102a and Agilent-MSD-TOF-1069A spectrometers 41.

### 3.2. Typical Procedure for the Synthesis of Mono- and Dibenzylated 1,2,3-triazoles from Aniline

#### 3.2.1. Benzyl Chloride

To a 20 mL round-bottomed flask equipped with a magnetic stirrer, were charged Cu(OAc)_2_·H_2_O (11 mg, 0.057 mmol, 5 mol%), 1,10-phenanthroline monohydrate (10 mg, 0.057 mmol, 5 mol%), and sodium L-ascorbate (224 mg, 1.14 mmol). After addition of a EtOH/H_2_O mixture (4:1 *v*/v, 7 mL), the resulting suspension was stirred for five minutes at room temperature. Subsequently, **I** (150 mg, 1.14 mmol), sodium azide (90 mg, 1.40 mmol), and benzyl chloride (0.16 mL, 1.40 mmol) were added to the reaction mixture, which was stirred during 18 h at room temperature. H_2_O (5 mL) was added to the reaction mixture and the precipitate was filtered off, washed thoroughly with H_2_O, petroleum ether, and dried under vacuum. The crude mixture containing mono- and dibenzylated products was separated by column chromatography and characterized as follows:

*N-Benzyl-N-((1-benzyl-1H-1,2,3-triazol-4-yl-)methyl)benzenamine* (**1a**). Using CH_2_Cl_2_ as eluent, the first fraction affords 169 mg (42% Yield) of **1a** as a white solid. mp 104–106 °C. ^1^H-NMR (CDCl_3_): δ = 4.63 (s, 2H, ArC*H*_2_N), 4.64 (s, 2H, ArC*H*_2_N_triazole_), 5.55 (s, 2H, NC*H*_2_C=C), 6.59 (t, 7.9 Hz, 1H, CH_ar_), 6.77 (d, 7.8 Hz, 2H, CH_ar_), 7.07–7.11 (m, 2H, CH_ar_), 7.19–7.23 (m, 5H, CH_ar_), 7.24–7.28 (m, 2H, CH_ar_), 7.30–7.36 (m, 3H, CH_ar_), 7.99 (s, 1H, CH_triazole_). ^13^C-NMR (CDCl_3_): δ = 45.9 (Ar*C*H_2_N_triazole_), 52.6 (Ar*C*H_2_N), 53.8 (N*C*H_2_C=C), 112.5 (CH_ar_), 116.2 (CH_ar_), 123.1 (*C*H_triazole_), 126.5 (CH_ar_), 126.6 (CH_ar_), 127.6 (CH_ar_), 127.9 (CH_ar_), 128.3 (CH_ar_), 128.6 (CH_ar_), 128.8 (CH_ar_), 136.0 (C_ar_), 138.9 (C_ar_), 144.7 (*C*_triazole_), 147.9 (C_ar_). FT-IR/ATR ν_max_ cm^−^^1^: 3116, 3084, 3064, 3027, 2960, 2920, 2885, 1726, 1596, 1527, 1506, 1494, 1450. HRMS (ESI-TOF) calculated for C_23_H_22_N_4_+H^+^: 355.1923; Found: 355.1908.

*N-((1-Benzyl-1H-1,2,3-triazol-4-yl-)methyl)benzenamine* (**2a**). Using CH_2_Cl_2_-MeOH (99:1) as eluent, the second fraction affords 105 mg (35% Yield) of **2a** as a white solid. mp 75–77 °C. ^1^H-NMR (DMSO-*d*_6_): δ = 4.29 (d, 5.4 Hz, 2H, NC*H*_2_C=C), 5.57 (s, 2H, ArC*H*_2_N), 6.01 (t, 6.1 Hz, 1H, N*H*), 6.54 (t, 7.7 Hz, 1H, CH_ar_), 6.61 (d, 7.6 Hz, 2H, CH_ar_), 7.05–7.08 (m, 2H, CH_ar_), 7.28–7.36 (m, 5H, CH_ar_), 7.99 (s, 1H, CH_triazole_). ^13^C-NMR (DMSO-*d*_6_): δ = 39.1 (Ar*C*H_2_N), 53.2 (N*C*H_2_C=C), 112.8 (CH_ar_), 116.5 (CH_ar_), 123.3 (*C*H_triazole_), 128.3 (CH_ar_), 128.5 (CH_ar_), 129.2 (CH_ar_), 129.3 (CH_ar_), 136.6 (C_ar_), 146.5 (*C*_triazole_), 148.8 (C_ar_). FT-IR/ATR ν_max_ cm^−^^1^: 3401, 3104, 3058, 2950, 2920, 2893, 2848, 1599, 1507, 1493, 1454, 1439. HRMS (ESI-TOF) calculated for C_16_H_16_N_4_+H^+^: 264.1453; Found: 264.1459.

#### 3.2.2. 4-Fluorobenzyl Chloride

The general procedure was followed using 1.40 mmol 4-fluorobenzyl chloride. The mixture of products was purified by column chromatography and characterized as follows:

*N-(4-Fluorobenzyl)-N-((1-(4-fluorobenzyl)-1H-1,2,3-triazol-4-yl-)methyl)benzenamine* (**1b**). Using CH_2_Cl_2_ as eluent, the first fraction affords 178 mg (40% yield) of **1b** as a white solid. mp 93–95 °C. ^1^H-NMR (CDCl_3_): δ = 4.60 (s, 2H, ArC*H*_2_N), 4.63 (s, 2H, ArC*H*_2_N_triazole_), 5.54 (s, 2H, NC*H*_2_C=C), 6.61 (t, 7.8 Hz, 1H, CH_ar_), 6.76 (d, 7.8 Hz, 2H, CH_ar_), 7.07–7.10 (m, 4H, CH_ar_), 7.15–7.17 (m, 2H, CH_ar_), 7.24–7.27 (m, 2H, CH_ar_), 7.30–7.33 (m, 2H, CH_ar_), 7.99 (s, 1H, CH_triazole_). ^13^C-NMR (CDCl_3_): δ = 45.8 (Ar*C*H_2_N_triazole_), 51.8 (Ar*C*H_2_N), 53.1 (N*C*H_2_C=C), 112.6 (CH_ar_), 115.0 (d, 21.3 Hz, CH_ar-F_), 115.4 (d, 21.4 Hz, CH_ar-F_), 116.4 (CH_ar_), 123.0 (*C*H_triazole_), 128.4 (d, 8.8 Hz, CH_ar-F_), 128.8 (CH_ar_), 130.0 (d, 8.8 Hz, CH_ar-F_), 132.2 (d, 2.5 Hz, C_ar-F_), 134.9 (d, 2.5 Hz, C_ar-F_), 144.6 (*C*_triazole_), 147.8 (C_ar_), 160.4 (d, 241.2 Hz, C_ar-F_), 162.3 (d, 243.6 Hz, C_ar-F_). FT-IR/ATR ν_max_ cm^−^^1^: 3108, 3064, 2857, 2927, 2859, 1725, 1594, 1504, 1456, 1442, 1389. HRMS (ESI-TOF) calculated for C_23_H_20_F_2_N_4_+H^+^: 391.1734; Found: 391.1720.

*N-((1-(4-Fluorobenzyl)-1H-1,2,3-triazol-4-yl-)methyl)benzenamine* (**2b**). Using CH_2_Cl_2_:MeOH (99:1) as eluent, the second fraction affords 100 mg (31% yield) of **2b** as a white solid. mp 88–90 °C. ^1^H-NMR (DMSO-*d*_6_): δ = 4.27 (d, 5.4 Hz, 2H, NC*H*_2_C=C), 5.56 (s, 2H, ArC*H*_2_N), 6.01 (t, 6.1 Hz, 1H, N*H*), 6.54 (t, 7.9 Hz, 1H, CH_ar_), 6.63 (d, 7.8 Hz, 2H, CH_ar_), 7.05–7.08 (m, 2H, CH_ar_), 7.17–7.21 (m, 2H, CH_ar_), 7.35–7.38 (m, 2H, CH_ar_), 8.00 (s, 1H, CH_triazole_). ^13^C-NMR (DMSO-*d*_6_): δ = 39.1 (Ar*C*H_2_N), 52.3 (N*C*H_2_C=C), 112.8 (CH_ar_), 115.9 (d, 21.3 Hz, CH_ar-F_), 116.5 (CH_ar_), 123.2 (*C*H_triazole_), 129.3 (CH_ar_), 130.6 (d, 8.8 Hz, CH_ar-F_), 132.9 (d, 3.8 Hz, C_ar-F_), 146.6 (C_triazole_), 148.9 (C_ar_), 162.3 (d, 243 Hz, C_ar-F_). FT-IR/ATR ν_max_ cm^−^^1^: 3397, 3105, 3058, 3048, 2952, 2920, 2893, 2846, 1719, 1599, 1506, 1459, 1439. HRMS (ESI-TOF) calculated for C_16_H_15_FN_4_+H^+^: 283.1359; Found: 283.1364.

#### 3.2.3. 4-Chlorobenzyl Chloride

The general procedure was followed using 1.40 mmol of 4-chlorobenzyl chloride. The mixture of products was purified by column chromatography and characterized as follows:

*N-(4-Chlorobenzyl)-N-((1-(4-chlorobenzyl)-1H-1,2,3-triazol-4-yl-)methyl)benzenamine* (**1c**). Using CH_2_Cl_2_ as eluent, the first fraction affords 188 mg (39% yield) of **1c** as a white solid. mp 126–128 °C. ^1^H-NMR (CDCl_3_): δ = 4.61 (s, 2H, ArCH_2_N), 4.65 (s, 2H, ArCH_2_N_triazole_), 5.55 (s, 2H, NCH_2_C=C), 6.61 (t, 7.7 Hz, 1H, CH_ar_), 6.75 (d, 7.8 Hz, 2H, CH_ar_), 7.09–7.12 (m, 2H, CH_ar_), 7.22–7.25 (m, 4H, CH_ar_), 7.32 (d, 7.8 Hz, 2H, CH_ar_), 7.40 (d, 7.7 Hz, 2H, CH_ar_), 8.02 (s, 1H, CH_triazole_). ^13^C-NMR (CDCl_3_): δ = 45.9 (ArCH_2_N_triazole_), 51.8 (ArCH_2_N), 53.2 (NCH_2_C=C), 112.6 (CH_ar_), 116.4 (CH_ar_), 123.1 (CH_triazole_), 128.2 (CH_ar_), 128.4 (CH_ar_), 128.6 (CH_ar_), 128.8 (CH_ar_), 129.6 (CH_ar_), 131.1 (C_ar_), 132.7 (C_ar_), 135.0 (C_ar_), 138.0 (C_ar_), 144.6 (C_triazole_), 147.7 (C_ar_). FT-IR/ATR ν_max_ cm^−^^1^: 3129, 3085, 3064, 3041, 2925, 1758, 1597, 1575, 1505, 1491, 1458, 1435. HRMS (ESI-TOF) calculated for C_23_H_20_Cl_2_N_4_+H^+^: 423.1143; Found: 423.1142

*N-((1-(4-Chlorobenzyl)-1H-1,2,3-triazol-4-yl-)methyl)benzenamine* (**2c**). Using CH_2_Cl_2_:MeOH (99:1) as eluent, the second fraction affords 119 mg (35% yield) of **2c** as a white solid. mp 83–85 °C. ^1^H-NMR (DMSO-*d*_6_): δ = 4.29 (d, 5.4 Hz, 2H, NCH_2_C=C), 5.57 (s, 2H, ArCH_2_N), 6.03 (bs, 1H, NH), 6.54 (t, 7.9 Hz, 1H, CH_ar_), 6.64 (d, 7.8 Hz, 2H, CH_ar_), 7.05–7.08 (m, 2H, CH_ar_), 7.31 (d, 7.7 Hz, 2H, CH_ar_), 7.42 (d, 7.8 Hz, 2H, CH_ar_), 8.01 (s, 1H, CH_triazole_). ^13^C-NMR (DMSO-*d*_6_): δ = 39.1 (ArCH_2_N), 52.4 (NCH_2_C=C), 112.8 (CH_ar_), 116.5 (CH_ar_), 123.4 (CH_triazole_), 129.1 (CH_ar_), 129.3 (CH_ar_), 130.2 (CH_ar_), 133.2 (C_ar_), 135.7 (C_ar_), 146.6 (C_triazole_), 148.8 (C_ar_). FT-IR/ATR ν_max_ cm^−^^1^: 3427, 3322, 3123, 3066, 3046, 2947, 2917, 2849, 1600, 1509, 1490, 1458, 1427, 1409. HRMS (ESI-TOF) calculated for C_16_H_15_ClN_4_+H^+^: 299.1019; Found: 299.1063.

#### 3.2.4. 4-Bromobenzyl Chloride

The general procedure was followed using (1.40 mmol) of 4-bromobenzyl chloride. The mixture of products was purified by column chromatography and characterized as follows:

*N-(4-Bromobenzyl)-N-((1-(4-bromobenzyl)-1H-1,2,3-triazol-4-yl-)methyl)benzenamine* (**1d**). Using CH_2_Cl_2_ as eluent, the first fraction affords 245 mg (42% yield) of **1d** as a white solid. mp 131–133 °C. ^1^H-NMR (CDCl_3_): δ = 4.59 (s, 2H, ArCH_2_N), 4.65 (s, 2H, ArCH_2_N_triazole_), 5.54 (s, 2H, NCH_2_C=C), 6.61 (t, 7.8 Hz, 1H, CH_ar_), 6.75 (d, 7.8 Hz, 2H, CH_ar_), 7.09–7.12 (m, 2H, CH_ar_), 7.16–7.20 (m, 4H, CH_ar_), 7.46 (d, 7.8 Hz, 2H, CH_ar_), 7.55 (d, 7.7 Hz, 2H, CH_ar_), 8.02 (s, 1H, CH_triazole_). ^13^C-NMR (CDCl_3_): δ = 46.0 (ArCH_2_N_triazole_), 51.9 (ArCH_2_N), 53.3 (NCH_2_C=C), 112.7 (CH_ar_), 116.4 (CH_ar_), 119.5 (Ca_r_), 121.3 (C_ar_), 123.2 (C_triazole_), 128.8 (CH_ar_), 128.9 (CH_ar_), 129.9 (CH_ar_), 131.2 (CH_ar_), 131.6 (CH_ar_), 135.5 (C_ar_), 138.5 (C_ar_), 144.7 (C_triazole_), 147.7 (C_ar_). FT-IR/ATR ν_max_ cm^−^^1^: 3139, 3043, 3012, 2977, 2911, 2935, 2800, 1596, 1515, 1499, 1436, 1425. HRMS (ESI-TOF) calculated for C_23_H_20_Br_2_N_4_+H^+^: 513.0133; Found: 513.0146.

*N-((1-(4-Bromobenzyl)-1H-1,2,3-triazol-4-yl-)methyl)benzenamine* (**2d**). Using CH_2_Cl_2_:MeOH (99:1) as eluent, the second fraction affords 113 mg (29% yield) of 2d as a white solid. mp 114–116 °C. ^1^H-NMR (DMSO-*d*_6_): δ = 4.29 (d, 5.4 Hz, 2H, NCH_2_C=C), 5.56 (s, 2H, ArCH_2_N), 6.01 (t, 5.4 Hz, 1H, NH), 6.55 (t, 7.8 Hz, 1H, CH_ar_), 6.62 (d, 7.8 Hz, 2H, CH_ar_), 7.05–7.08 (m, 2H, CH_ar_), 7.24 (d, 7.7 Hz, 2H, CH_ar_), 7.56 (d, 7.8 Hz, 2H, CH_ar_), 8.00 (s, 1H, CH_triazole_). ^13^C-NMR (DMSO-d_6_): δ = 39.0 (ArCH_2_N), 52.4 (NCH_2_C=C), 112.8 (CH_ar_), 116.5 (CH_ar_), 121.8 (C_ar_), 123.4 (CH_triazole_), 129.3 (CH_ar_), 130.6 (CH_ar_), 132.1 (CH_ar_), 136.1 (C_ar_), 146.6 (C_triazole_), 148.8 (C_ar_). FT-IR/ATR ν_max_ cm^−^^1^: 3492, 3398, 3320, 3123, 3045, 2944, 2887, 1600, 1510, 1487, 1460, 1426. HRMS (ESI-TOF) calculated for C_16_H_15_BrN_4_+H^+^: 344.0460; Found: 344.0465.

#### 3.2.5. 4-Iodobenzyl Bromide

The general procedure was followed using (1.40 mmol) of 4-iodobenzyl bromide. The mixture of products was purified by column chromatography and characterized as follows:

*N-(4-Iodobenzyl)-N-((1-(4-iodobenzyl)-1H-1,2,3-triazol-4-yl-)methyl)benzenamine* (**1e**). Using CH_2_Cl_2_ as eluent, the first fraction affords 172 mg (25% yield) of **1e** as a white solid. mp 138–140 °C. ^1^H-NMR (CDCl_3_): δ = 4.57 (s, 2H, ArCH_2_N), 4.64 (s, 2H, ArCH_2_N_triazole_), 5.51 (s, 2H, NCH_2_C=C), 6.61 (t, 7.8 Hz, 1H, CH_ar_), 6.74 (d, 7.8 Hz, 2H, CH_ar_), 7.02–7.04 (m, 2H, CH_ar_), 7.08–7.11 (m, 4H, CH_ar_), 7.63 (d, 7.8 Hz, 2H, CH_ar_), 7.71 (d, 7.7 Hz, 2H, CH_ar_), 8.01 (s, 1H, CH_triazole_). ^13^C-NMR (CDCl_3_): δ = 45.9 (ArCH_2_N_triazole_), 52.0 (ArCH_2_N), 53.4 (NCH_2_C=C), 92.1 (C_ar_), 94.2 (C_ar_), 112.6 (CH_ar_), 116.4 (CH_ar_), 123.2 (C_triazole_), 128.8 (CH_ar_), 129.0 (CH_ar_), 129.9 (CH_ar_), 135.8 (C_ar_), 137.0 (CH_ar_), 137.4 (CH_ar_), 138.9 (C_ar_), 144.6 (C_triazole_), 147.7 (C_ar_). FT-IR/ATR ν_max_ cm^−^^1^: 3130, 3062, 3024, 2955, 2922, 2905, 2855, 1597, 1504, 1482, 1456, 1431. HRMS (ESI-TOF) calculated for C_23_H_20_I_2_N_4_+H^+^: 606.9855; Found: 606.9856.

*N-((1-(4-Iodobenzyl)-1H-1,2,3-triazol-4-yl-)methyl)benzenamine* (**2e**). Using CH_2_Cl_2_:MeOH (99:1) as eluent, the second fraction affords 195 mg (42% yield) of **2e** as a white solid. mp 128–130 °C. ^1^H-NMR (DMSO-*d*_6_): δ = 4.27 (d, 5.4 Hz, 2H, NCH_2_C=C), 5.52 (s, 2H, ArCH_2_N), 5.99 (t, 5.4 Hz, 1H, NH), 6.54 (t, 7.8 Hz, 1H, CH_ar_), 6.62 (d, 7.8 Hz, 2H, CH_ar_), 7.04–7.07 (m, 2H, CH_ar_), 7.08 (d, 7.7 Hz, 2H, CH_ar_), 7.72 (d, 7.8 Hz, 2H, CH_ar_), 7.98 (s, 1H, CH_triazole_). ^13^C-NMR (DMSO-*d*_6_): δ = 38.6 (ArCH_2_N), 52.4 (NCH_2_C=C), 94.2 (C_ar_), 112.3 (CH_ar_), 116.0 (CH_ar_), 122.9 (CH_triazole_), 128.8 (CH_ar_), 130.1 (CH_ar_), 135.9 (C_ar_), 137.4 (C_ar_), 146.1 (C_triazole_), 148.3 (C_ar_). FT-IR/ATR ν_max_ cm^−^^1^: 3452, 3390, 3303, 3121, 3039, 2947, 2854, 1598, 1504, 1483, 1458, 1428. HRMS (ESI-TOF) calculated for C_16_H_15_IN_4_+H^+^: 391.0375; Found: 391.0420.

### 3.3. Typical Procedure for the Selective Synthesis of Dibenzylated 1,2,3-triazoles Derived from Aniline

To a 20 mL round-bottomed flask equipped with a magnetic stirrer, were charged Cu(OAc)_2_·H_2_O (11 mg, 0.057 mmol, 5 mol%), 1,10-phenanthroline monohydrate (10 mg, 0.057 mmol, 5 mol%), and sodium L-ascorbate (224 mg, 1.14 mmol). After addition of a mixture of EtOH/H_2_O (7 mL, 4:1 *v*/*v*), the resulting suspension was stirred for five minutes at room temperature. Subsequently, **I** (150 mg, 1.14 mmol), sodium azide (90 mg, 1.40 mmol), and benzyl chloride (0.32 mL, 2.80 mmol) were added to the reaction mixture, which was stirred during 24 h at room temperature. H_2_O (5 mL) was added to the reaction mixture and the precipitate was filtered off, washed thoroughly with H_2_O, petroleum ether, and dried under vacuum. The dibenzylated products **1a**–**e** were purified by column chromatography using CH_3_Cl as eluent.

### 3.4. Reaction of N-((1-(4-Iodobenzyl)-1H-1,2,3-triazol-4-yl-)methyl)benzenamine (**2e**) with p-Halogenated Benzyl Chlorides

*N-(4-Fluorobenzyl)-N-((1-(4-iodobenzyl)-1H-1,2,3-Triazol-4-yl-)methyl)benzenamine* (**3**). In a 20 mL round-bottomed flask equipped with a magnetic stirrer, *p*-fluorobenzyl chloride (33 μL, 0.281 mmol) was added to a solution of **2e** (100 mg, 0.256 mmol) in CH_2_Cl_2_ (10 mL) and the reaction mixture was allowed to stir for 12 h at 40 °C. The mixture was dried under vacuum and the residue was purified by column chromatography using CH_3_Cl as eluent to give 114 mg (90% yield) of the title product as a waxy solid; mp 86–88 °C. ^1^H-NMR (CDCl_3_): δ = 4.56 (s, 2H, ArC*H*_2_N), 4.68 (s, 2H, ArC*H*_2_N_triazole_), 5.41 (s, 2H, NC*H*_2_C=C), 6.76 (t, 7.8 Hz, 1H, CH_ar_), 6.78 (d, 7.8 Hz, 2H, CH_ar_), 6.95 (d, 7.8 Hz, 2H, CH_ar_), 6.99 (d, 7.7 Hz, 2H, CH_ar_), 7.19–7.21 (m, 4H, CH_ar_), 7.27 (s, 1H, CH_triazole_), 7.71 (d, 7.7 Hz, 2H, CH_ar_). ^13^C-NMR (CDCl_3_): δ = 46.6 (Ar*C*H_2_N_triazole_), 53.5 (Ar*C*H_2_N), 54.0 (N*C*H_2_C=C), 94.5 (C_ar_), 113.2 (CH_ar_), 115.4 (d, 20.1 Hz, CH_ar-F_), 117.6 (CH_ar_), 121.6 (*C*H_triazole_), 128.4 (d, 8.8 Hz, CH_ar-F_), 129.3 (CH_ar_), 129.6 (CH_ar_), 134.0 (d, 2.6 Hz, C_ar-F_), 134.3 (C_ar_), 138.2 (CH_ar_), 146.1 (C_triazole_), 148.3 (C_ar_), 161.9 (d, 263 Hz, C_ar-F_). FT-IR/ATR ν_max_ cm^−^^1^: 3119, 3075, 3037, 3023, 2979, 2917, 1729, 1594, 1503, 1484, 1458, 1440. HRMS (ESI-TOF) calculated for C_23_H_20_IFN_4_+H^+^: 499.0717; Found: 499.0788.

*N-(4-Iodoobenzyl)-N-((1-(4-iodobenzyl)-1H-1,2,3-Triazol-4-yl-)methyl)benzenamine* (**1e**). The above procedure was followed using 4-iodobenzyl chloride (0.281 mmol). The title product (146 mg, 94% yield) was obtained as a waxy solid after purification by column chromatography using CH_3_Cl as eluent.

## 4. Conclusions

In conclusion, we have reported a one-pot methodology for the synthesis of a series of mono-, and dibenzylated 1,2,3-triazoles utilizing “click” chemistry. The selective preparation of dibenzylated triazoles **1a**–**e** was achieved by addition of excess of *p*-halogenated benzyl derivatives in a pseudo-four component click process. Particularly appealing is the reactivity of the available N-H bond in the *N*-benzylated triazoles **2a**–**e** which may be tunable with a variety of functional groups. The possible applications of the synthetized 1,2,3-triazoles are the topic of current investigation in our laboratory.
